# Fucoxanthin Inhibits the Proliferation and Metastasis of Human Pharyngeal Squamous Cell Carcinoma by Regulating the PI3K/Akt/mTOR Signaling Pathway

**DOI:** 10.3390/molecules29153603

**Published:** 2024-07-30

**Authors:** Hao-Fei Du, Jia-Min Jiang, Si-Han Wu, Yan-Fang Shi, Hai-Tian Liu, Zheng-Hao Hua, Cai-Sheng Wang, Guo-Ying Qian, Hao-Miao Ding

**Affiliations:** Hwamei College of Life and Health Sciences, Zhejiang Wanli University, Ningbo 315100, China; youzanzizwu@163.com (H.-F.D.); jiangjiaminzwu@163.com (J.-M.J.); test9527wsh@163.com (S.-H.W.); fang34272024@163.com (Y.-F.S.); lht041207@163.com (H.-T.L.); huazh0214@163.com (Z.-H.H.); wangcs0528@163.com (C.-S.W.)

**Keywords:** fucoxanthin, human pharyngeal squamous cell carcinoma, proliferation, metastatic, PI3K/AKT/mTOR pathway, MMP−2/9

## Abstract

Human pharyngeal squamous cell carcinoma (HPSCC) is the most common malignancy in the head and neck region, characterized by high mortality and a propensity for metastasis. Fucoxanthin, a carotenoid isolated from brown algae, exhibits pharmacological properties associated with the suppression of tumor proliferation and metastasis. Nevertheless, its potential to inhibit HPSCC proliferation and metastasis has not been fully elucidated. This study represents the first exploration of the inhibitory effects of fucoxanthin on two human pharyngeal squamous carcinoma cell lines (FaDu and Detroit 562), as well as the mechanisms underlying those effects. The results showed dose-dependent decreases in the proliferation, migration, and invasion of HPSCC cells after fucoxanthin treatment. Further studies indicated that fucoxanthin caused a significant reduction in the expression levels of proteins in the phosphoinositide 3−kinase (PI3K)/protein kinase B (AKT)/mechanistic target of rapamycin (mTOR) pathway, as well as the downstream proteins matrix metalloproteinase (MMP)−2 and MMP−9. Specific activators of PI3K/AKT reversed the effects of fucoxanthin on these proteins, as well as on cell proliferation and metastasis, in FaDu and Detroit 562 cells. Molecular docking assays confirmed that fucoxanthin strongly interacted with PI3K, AKT, mTOR, MMP−2, and MMP−9. Overall, fucoxanthin, a functional food component, is a potential therapeutic agent for HPSCC.

## 1. Introduction

Human pharyngeal squamous cell carcinoma (HPSCC), a malignant tumor originating in the head and neck region, is characterized by high invasiveness and metastatic potential; it constitutes approximately 3–5% of all head and neck cancers [1]. Because of its anatomical proximity to the larynx, HPSCC often remains undetected until it reaches an advanced stage, resulting in a poor prognosis and a 5-year survival rate of approximately 40% [2,3]. Conventional treatment modalities for HPSCC involve surgical intervention combined with chemotherapy or radiotherapy. However, the complex anatomical structures of the head and neck lead to surgical challenges that hinder complete tumor eradication under conditions of local invasion and metastasis [4]. Procedures such as total laryngectomy or partial laryngectomy, along with hypopharyngeal resection, can result in the functional loss of the corresponding organ and a significant decrease in quality of life. Although chemotherapy preserves laryngeal function, its clinical efficacy is limited by drug resistance and severe side effects, as well as a high recurrence rate [5]. Consequently, there is an urgent need to identify novel anti-HPSCC agents with high activity and low toxicity.

**Figure 1:**
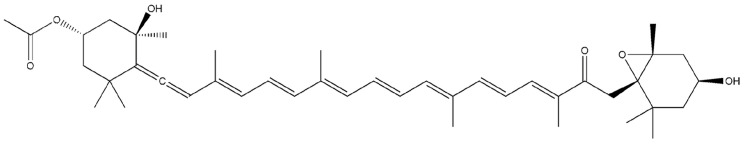
Chemical structure of fucoxanthin.

Fucoxanthin is a carotenoid present in edible brown algae, such as wakame and kelp, where it constitutes approximately 10% of the total marine carotenoid content. It is composed of a propenyl group, nine unconjugated double bonds, a 5,6-epoxy group, and several other oxygen-containing functional groups (Figure 1) [6]. Initial studies demonstrated that fucoxanthin possesses diverse pharmacological activities, including anti-obesity [7], antioxidant [8], anti-inflammatory [9], and anti-tumor effects [10]. Moreover, fucoxanthin is a safe, natural pigment-like compound [11,12] that has extensive applications in functional foods and health products. The anticancer (e.g., anti-metastatic and pro-apoptotic) effects of fucoxanthin have been demonstrated in various cancer cell lines, including lung, colon, ovarian, and breast cancer [13]. A breast cancer-focused study showed that fucoxanthin was able to exert anti-proliferative and anti-migratory effects by suppressing the malignant phenotype of MDA−MB−231 cells and reducing tumor-induced lymphangiogenesis [14]. There is increasing evidence that fucoxanthin has anti-metastatic and pro-apoptotic effects on lung cancer cells. These effects are mediated by inhibiting epithelial-mesenchymal transition and the phosphoinositide 3−kinase (PI3K)/protein kinase B (AKT)/NF(nuclear factor)−κB signaling pathway [15]. Studies thus far have provided important evidence to support further investigation of the roles of natural products in cancer. However, it is unclear whether fucoxanthin can prevent or decrease the proliferation and metastasis of HPSCC.

This study investigated the anti-proliferative, anti-migratory, and anti-invasive effects of fucoxanthin on FaDu and Detroit 562 HPSCC cells, which represent common and aggressive histopathological subtypes of HPSCC. The results showed that fucoxanthin can inhibit the proliferation, migration, invasion, and cell cycle progression of FaDu and Detroit 562 cells by modulating the PI3K/AKT/mechanistic target of the rapamycin (mTOR) signaling pathway and regulating the expression of the downstream proteins matrix metalloproteinases (MMP)−2 and MMP−9. Additionally, molecular docking analyses revealed low binding energies and high affinities between fucoxanthin and several proteins: PI3K, AKT, mTOR, MMP−2, and MMP−9. These findings provide important insights for screening anti-HPSCC drugs and establish a theoretical foundation for the development of therapeutic agents based on marine algae resources, as well as for the prevention and treatment of tumor diseases.

## 2. Results

### 2.1. Inhibitory Effects of Fucoxanthin on HPSCC Cells

**Figure 2:**
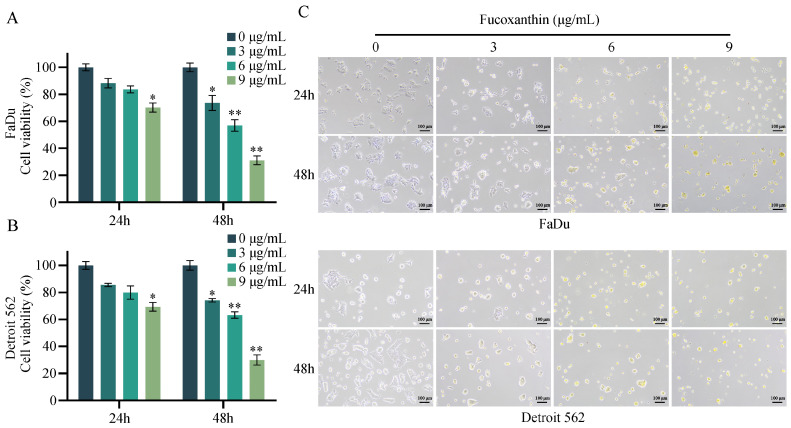
Fucoxanthin exhibits significant inhibitory effects on FaDu and Detroit 562 cells. The MTT assay was used to measure the viability of (**A**) FaDu and (**B**) Detroit 562 cells after 24 and 48 h of treatment with various concentrations of fucoxanthin. (**C**) Morphological changes in FaDu and Detroit 562 cells after treatment with various concentrations of fucoxanthin. * *p* < 0.05, ** *p* < 0.01.

HPSCC cell lines were treated with various concentrations of fucoxanthin (0, 3, 6, or 9 μg/mL) for 24 or 48 h. Subsequently, cell viability was assessed using the 3-(4,5-dimethylthiazol-2yl)-2,5 diphenyl tetrazolium bromide (MTT) assay. As shown in Figure 2A,B, fucoxanthin significantly reduced the viability of FaDu and Detroit 562 cells in a dose-dependent manner within 48 h. According to optical density values at 570 nm, we determined that the half-maximal inhibitory concentration (IC_50_) values of fucoxanthin for FaDu and Detroit 562 cells after 24 h were 17.91 μg/mL and 18.58 μg/mL, respectively. After 48 h, the IC_50_ values were 6.21 μg/mL for FaDu cells and 6.55 μg/mL for Detroit 562 cells. Importantly, fucoxanthin did not significantly affect the growth of normal cell lines. Inhibitory effects on normal cells were only observed after 48 h of treatment and at concentrations exceeding 12 μg/mL, with an IC_50_ value of 18.13 μg/mL (Figure S1). Therefore, we chose a 48-h fucoxanthin treatment duration for both FaDu and Detroit 562 cells. Subsequently, we used an inverted microscope to observe the morphology and number of cells treated with the four selected concentrations of fucoxanthin. As illustrated in Figure 2C, fucoxanthin altered cell morphologies and reduced cell numbers, particularly at higher concentrations, suggesting that fucoxanthin exhibits anti-proliferative and anti-migratory properties.

### 2.2. Fucoxanthin Inhibits the Proliferation of HPSCC Cells

**Figure 3:**
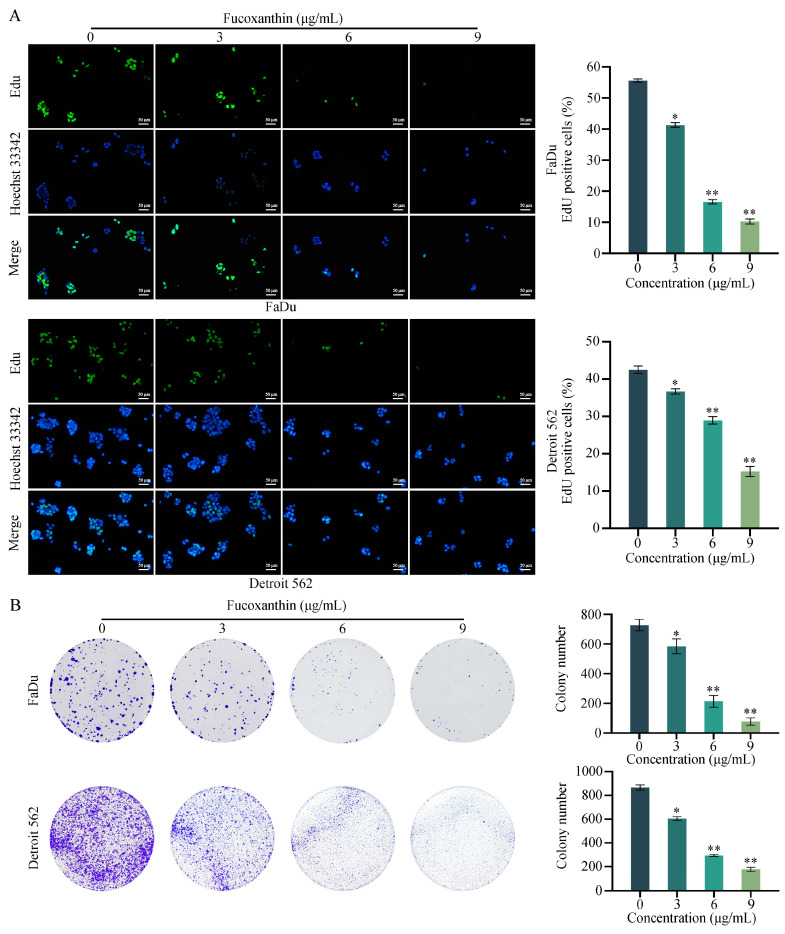
Effects of fucoxanthin on the proliferation abilities of FaDu and Detroit 562 cells. (**A**) EdU fluorescence microscopy was performed to qualitatively and quantitatively assess proliferation in FaDu and Detroit 562 cells after 48 h of treatment with fucoxanthin. (**B**) Colony formation assays were conducted to evaluate the effects of various concentrations of fucoxanthin on the colony-forming abilities of FaDu and Detroit 562 cells. * *p* < 0.05, ** *p* < 0.01.

To determine whether the reduced viability of FaDu and Detroit 562 cells was due to the proliferation-suppressing effects of fucoxanthin, we conducted EdU fluorescent staining experiments that assessed cell proliferation. The ratio of green fluorescent cells to blue fluorescent cells represented the proportion of proliferating cells among all cells. As shown in Figure 3A, in the absence of fucoxanthin, proliferating cells constituted 55.61% of the total FaDu cell population. After treatment with 0, 3, 6, and 9 μg/mL of fucoxanthin, this percentage decreased to 55.61%, 41.31%, 16.60%, and 10.30%, respectively. Similarly, in the absence of fucoxanthin, 45.26% of Detroit 562 cells were proliferating. After treatment with 0, 3, 6, and 9 μg/mL of fucoxanthin, the percentage of proliferating cells decreased to 34.27%, 14.78%, and 8.22%, respectively. The EdU experiment demonstrated that fucoxanthin can inhibit the proliferation of FaDu and Detroit 562 cells. Additionally, colony formation assays demonstrated that fucoxanthin significantly inhibited the colony-forming abilities of FaDu and Detroit 562 cells in a dose-dependent manner (Figure 3B). Overall, fucoxanthin exhibited strong proliferation-suppressing effects on FaDu and Detroit 562 cells.

### 2.3. Fucoxanthin Inhibits the Migration and Invasion of FaDu and Detroit 562 Cells

**Figure 4:**
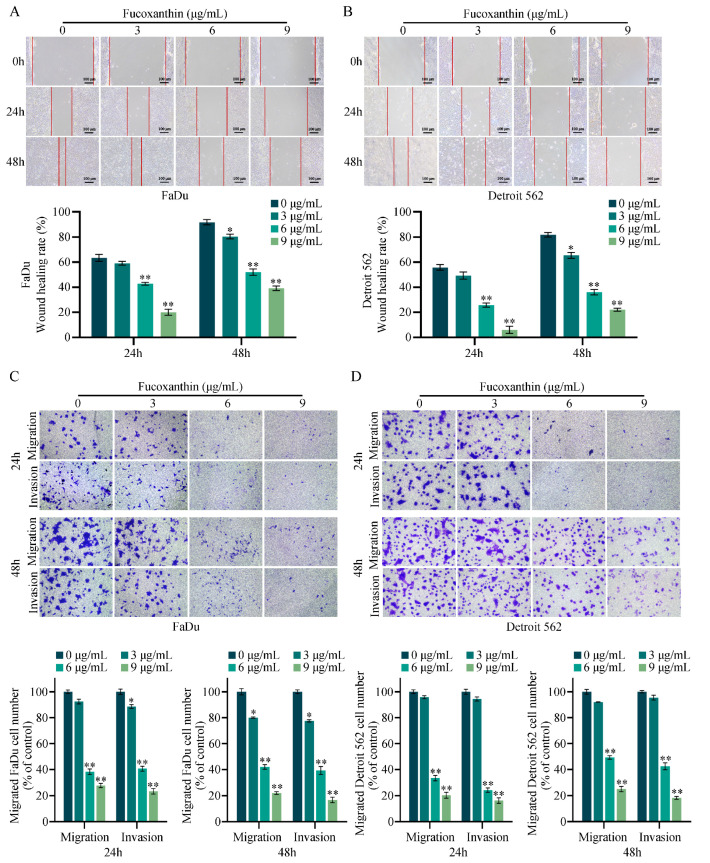
Fucoxanthin inhibits the migration and invasion of FaDu and Detroit 562 cells. (**A**,**B**) Treatment of FaDu and Detroit 562 cells with various concentrations of fucoxanthin for 24 and 48 h resulted in varying degrees of wound healing inhibition. (**C**,**D**) Transwell assays were conducted to assess the effects of fucoxanthin treatment for 24 and 48 h on the migration and invasion abilities of FaDu and Detroit 562 cells. * *p* < 0.05, ** *p* < 0.01.

After observing changes in cell morphology and proliferation ability, we examined the effects of fucoxanthin on the metastatic abilities of FaDu and Detroit 562 cells. As shown in Figure 4A,B, we created scratch wounds, then continued to treat the cells with various concentrations of fucoxanthin; a decrease in the wound closure rate was evident as the concentration of fucoxanthin increased. At 24 and 48 h, fucoxanthin-treated FaDu and Detroit 562 cells exhibited inferior wound healing compared with the control group, which displayed substantial closure. Transwell migration and invasion experiments yielded similar outcomes, demonstrating that fucoxanthin significantly reduced the metastatic abilities of FaDu and Detroit 562 cells at 24 and 48 h (Figure 4C,D). These results confirmed our hypothesis that fucoxanthin inhibits the metastatic activities of FaDu and Detroit 562 cells.

### 2.4. Fucoxanthin Induces Cell Cycle Arrest in HPSCC Cells

**Figure 5:**
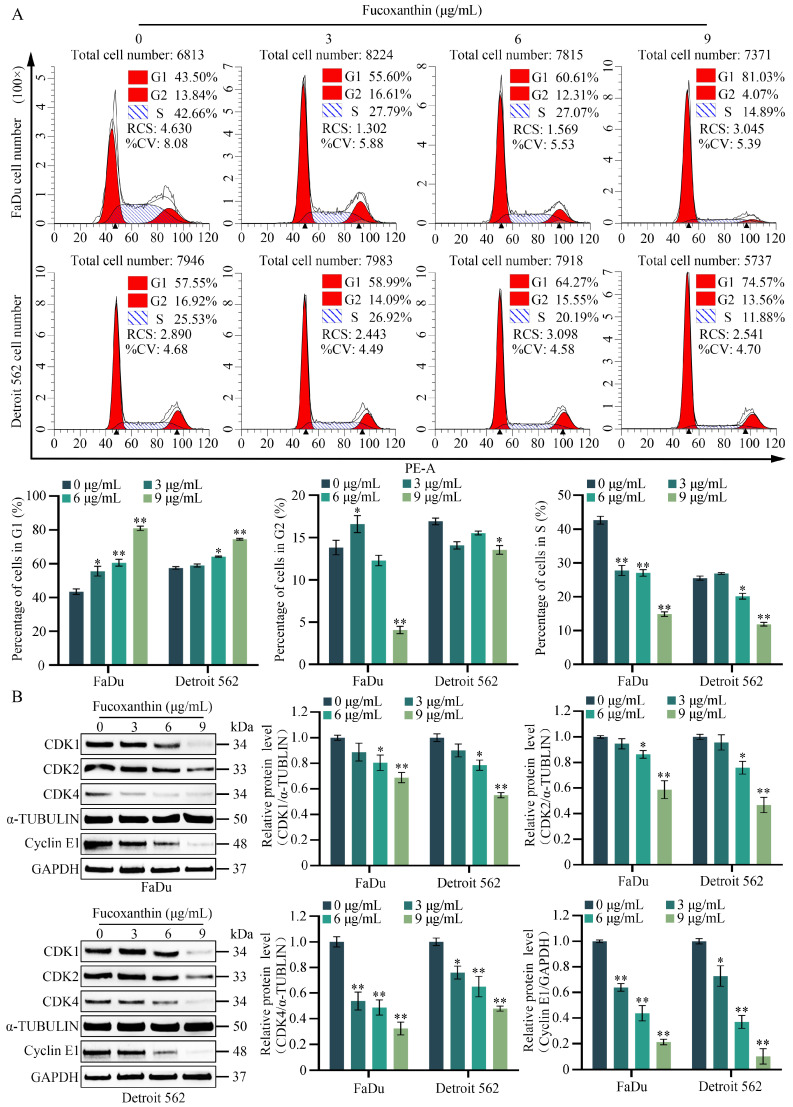
Fucoxanthin induces cell cycle arrest in FaDu and Detroit 562 cells. (**A**) Flow cytometry was performed to analyze the cell cycle distribution in fucoxanthin-treated FaDu and Detroit 562 cells after 48 h. (**B**) The protein expression levels of CDK1, CDK2, CDK4, and cyclin E1 were assessed in fucoxanthin-treated FaDu and Detroit 562 cells after 48 h. * *p* < 0.05, ** *p* < 0.01.

To further explore the inhibitory effects of fucoxanthin on the proliferation of FaDu and Detroit 562 cells, we performed flow cytometry with a focus on the cell cycle distribution. As indicated in Figure 5A, changes in cell cycle arrest were evident among FaDu and Detroit 562 cells that had been treated with various concentrations of fucoxanthin. As the concentration of fucoxanthin increased, the proportion of FaDu cells arrested in the G0/G1 phase substantially rose from 43.50% to 81.03%, suggesting that a substantial proportion of cells had been arrested in that phase. Concurrently, the percentage of cells in the S phase showed a decreasing trend, demonstrating that this effect of fucoxanthin was dose-dependent. Furthermore, treatment with 6 μg/mL and 9 μg/mL of fucoxanthin significantly suppressed cell cycle progression in Detroit 562 cells. Compared with untreated cells, the proportion of Detroit 562 cells arrested in the G0/G1 phase considerably increased from 54.55% to 74.57%. Based on these results, we further explored the effects of fucoxanthin on the cell cycle by conducting Western blotting analyses of cell cycle-related genes (including cyclin dependent kinase [CDK]1, CDK2, CDK4, and cyclin E1) in FaDu and Detroit 562 cells. The results showed that the expression levels of CDK1, CDK2, CDK4, and cyclin E1 decreased in a fucoxanthin dose-dependent manner (Figure 5B). These findings suggested that fucoxanthin induces cell cycle arrest in the G0/G1 phase within FaDu and Detroit 562 cell lines, thereby disrupting cell cycle progression.

### 2.5. Fucoxanthin Downregulates the Expression Levels of MMP−2, MMP−9, and PI3K/AKT/mTOR Signaling Pathway Components

**Figure 6:**
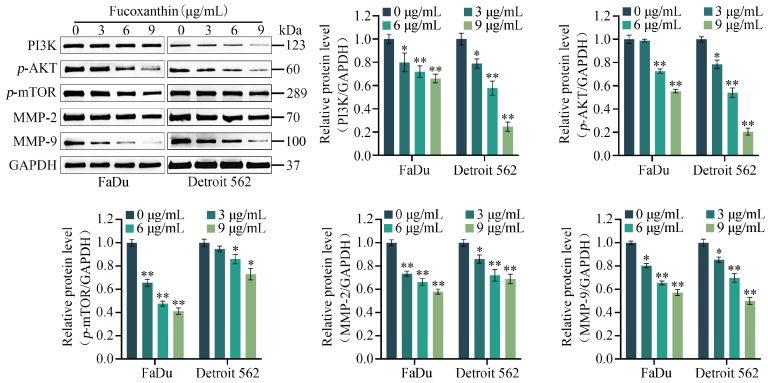
Changes in the PI3K/AKT/mTOR signaling pathway and the protein expression levels of MMP−2 and MMP−9 after FaDu and Detroit 562 cells had been treated with various concentrations of fucoxanthin for 48 h. * *p* < 0.05, ** *p* < 0.01.

To further characterize the inhibitory effects of fucoxanthin on the proliferation and metastasis of FaDu and Detroit 562 cells, we analyzed its effects on the PI3K/AKT/mTOR signaling pathway, as well as the downstream proteins MMP−2 and MMP−9. Western blotting analysis revealed that in FaDu and Detroit 562 cells, fucoxanthin treatment downregulated the protein levels of PI3K, *p*−AKT, and *p*−mTOR, but the protein levels of AKT and mTOR were not significantly altered (Figure S2). Additionally, concomitant downregulation of the downstream proteins MMP−2 and MMP−9 was evident, consistent with suppression of the PI3K/AKT/mTOR signaling pathway in these cells (Figure 6). In summary, fucoxanthin caused dose-dependent downregulation of PI3K, *p*−AKT, *p*−mTOR, MMP−2, and MMP−9 protein expression in FaDu and Detroit 562 cells.

### 2.6. 740 Y−P Reversed the Fucoxanthin-Induced Downregulation of PI3K/AKT/mTOR, MMP−2, and MMP−9 Protein Levels

**Figure 7:**
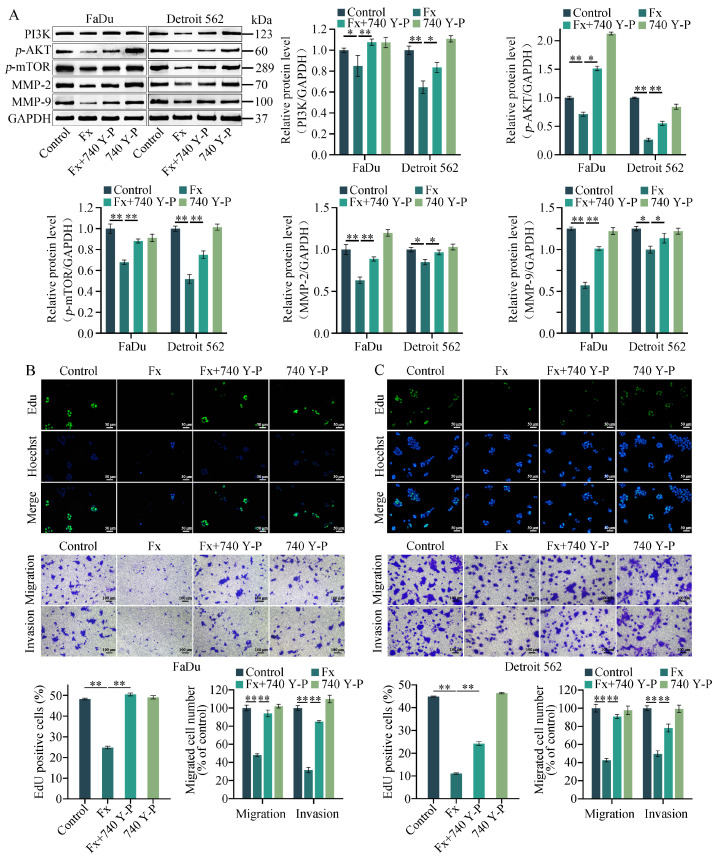
Rescue effects of the PI3K/AKT pathway activator, 740 Y−P. (**A**) Changes in the expression levels of PI3K/AKT/mTOR pathway-associated proteins, as well as MMP−2 and MMP−9, were assessed in FaDu and Detroit 562 cells after treatment with fucoxanthin for 48 h (Fx; 6 μg/mL) and 740 Y−P (50 μg/mL). The proliferation and metastasis of (**B**) FaDu and (**C**) Detroit 562 cells, after 48 h of treatment with Fx (6 μg/mL) and 740 Y−P (50 μg/mL), were evaluated with EdU and Transwell assays. * *p* < 0.05, ** *p* < 0.01.

To demonstrate the regulatory effects of fucoxanthin on the PI3K/AKT/mTOR pathway, the PI3K/AKT pathway activator 740 Y−P was used for reverse validation of the expression levels of proteins within the pathway. As revealed in Figure 7A, in the combined treatment group, the administration of 740 Y−P reversed the fucoxanthin-induced downregulation of PI3K, *p*−AKT, *p*−mTOR, MMP−2, and MMP−9 protein levels; AKT and mTOR protein expression levels did not significantly change during treatment with either of the two drugs (Figure S3). Furthermore, the effects of 740 Y−P treatment on cell proliferation and metastasis were separately validated by EdU and Transwell experiments. The results indicated that compared with single treatment, combined treatment with both drugs significantly enhanced the mean intensity of EdU green fluorescence and the number of migrating cells (Figure 7B,C). Thus, fucoxanthin exerted anti-proliferative and anti-metastatic effects by inhibiting the activation of the PI3K/AKT/mTOR pathway, thereby downregulating the expression levels of MMP−2 and MMP−9.

### 2.7. Interactions of Fucoxanthin with Target Proteins

**Figure 8:**
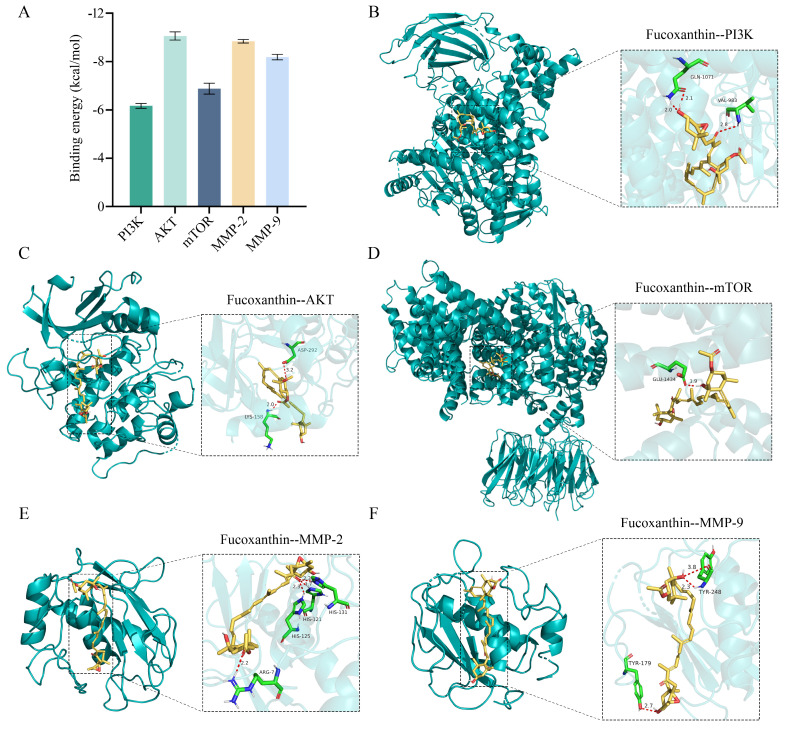
Molecular docking results of fucoxanthin with protein targets. (**A**) Binding energies of fucoxanthin and targets in molecular docking analyses. (**B**–**F**) Molecular docking between fucoxanthin and target proteins (PI3K, AKT, mTOR, MMP−2, and MMP−9). For clarity, only hydrogen bond-interacting residues are labeled. Hydrogen bonding interactions are represented by red dashed lines.

To further explore the interactions of fucoxanthin with pathway proteins, we conducted molecular docking simulations involving six key target proteins: PI3K, AKT, mTOR, MMP−2, and MMP−9. Molecular docking revealed that fucoxanthin exhibited binding energies of <−6 kcal/mol with all six target proteins (Figure 8A), indicating high binding efficiency. These interactions involved hydrogen bonds, salt bridges, hydrophobic interactions, and other forces (Tables S1–S13). Visual analyses of the fucoxanthin-protein complexes were performed using PyMOL 4.6 software. The results indicated a relatively stable interaction between fucoxanthin and PI3K, with a binding energy of −6.25 kcal/mol; key residues involved were GLN−1071 and VAL−983 (Figure 8B). The binding energy of fucoxanthin with AKT was −10.59 kcal/mol; key residues involved were ASP−292 and LYS−158. Similarly, the binding energy with mTOR was −7.32 kcal/mol, and the key residue involved was GLU−1434 (Figure 8C,D). MMP−2, an important protein in cell migration and invasion, showed a high binding affinity with fucoxanthin (−10.26 kcal/mol); key residues involved were HIS−131, HIS−121, HIS−125, and ARG−7 (Figure 8E). MMP−9 also exhibited a significant interaction with fucoxanthin (binding energy of approximately −9.28 kcal/mol); key residues involved were TRY−179 and TRY−248 (Figure 8F). Finally, fucoxanthin formed strong hydrogen bonds with residues at short distances (approximately 2.0 Å), contributing to the formation of stable complexes.

## 3. Discussion

Head and neck squamous cell carcinoma is the sixth most common cancer worldwide and primarily affects the mucosal epithelium of the oral cavity, pharynx, and larynx. HPSCC, one of the most common malignancies in the head and neck region, is typically attributed to factors such as excessive alcohol consumption and tobacco use [16]. The incidence of HPSCC is steadily increasing, with a projected 30% rise by 2030 (equivalent to 1.08 million new cases annually). In 2020 alone, there were approximately 84,000 cases of hypopharyngeal cancer worldwide, along with an estimated 39,000 deaths [17,18,19]. Surgical resection is the typical treatment for HPSCC in the oral cavity, followed by adjuvant radiotherapy or chemoradiotherapy depending on disease stage. However, conventional chemotherapy regimens heavily rely on synthetic drugs used for targeted applications, which may neither effectively prevent side effects nor the subsequent decrease in quality of life [20,21]. Additionally, targeted therapies are relatively expensive and thus unaffordable for many patients. In this context, the advantages of naturally derived drugs are evident because these natural molecules offer safer, more accessible, and more cost-effective options [22].

Fucoxanthin is commonly present in marine brown algae, but remains one of the least-studied natural compounds. Previous studies have indicated that fucoxanthin possesses various biological activities, including antioxidant properties, anti-inflammatory effects, and the potential to mitigate cardiovascular diseases [23,24,25]. Importantly, its anticancer activities have been demonstrated. A nasopharyngeal carcinoma-focused study showed that fucoxanthin downregulates autophagy-related proteins under stress conditions, thereby inducing apoptosis [26]. Jia et al. [27] discovered that fucoxanthin significantly inhibits the migration and invasion of lung cancer cells in vitro; it also enhances lung cancer cell sensitivity to gefitinib in vitro. Fucoxanthin has also been shown to inhibit the proliferation and migration of human breast cancer cells through multiple pathways [28]. Importantly, the safety of fucoxanthin has been confirmed. Beppu et al. [12] administered oral fucoxanthin at doses of 500 mg/kg and 1000 mg/kg to male and female ICR mice for 30 days; they observed no mortality or histological abnormalities in various organs. Additional animal toxicology studies have demonstrated the safety of fucoxanthin at doses of ≥200 mg/kg [29]. However, some in vitro studies have shown that high doses of fucoxanthin are toxic to human cells. For example, 10 μM fucoxanthin reduced the viability of human lymphocytes by 40% within 24 h; 40 μM fucoxanthin exhibited toxicity to keratinocytes within 16 h [30,31]. Consistent with these findings, our results indicate that within safe drug concentrations for normal cells, fucoxanthin inhibits the proliferation and cell cycle progression of FaDu and Detroit 562 cells, while also reducing the expression levels of cell cycle-related proteins. Additionally, wound healing and Transwell assays confirmed the inhibitory effects of fucoxanthin on the migration and invasion of FaDu and Detroit 562 cells. These results underscore the need to further explore the mechanisms by which fucoxanthin inhibits the proliferation and metastasis of HPSCC, potentially revealing new therapeutic approaches.

The PI3K/Akt/mTOR signaling pathway orchestrates a multifaceted network of intracellular events to precisely regulate the functions of numerous downstream proteins. This complex pathway is closely involved in critical cellular processes, such as survival, proliferation, migration, metabolism, and angiogenesis [32]. Aberrant activation of this pathway has been observed in a variety of human cancers, including head and neck cancer, leukemia, glioblastoma, and colorectal cancer [33,34,35]. It offers a promising target pathway for anticancer strategies based on natural compounds [36]. Fucoxanthin reportedly can inhibit the malignant proliferation of non-small cell lung cancer by inhibiting phosphorylation of the PI3K/AKT pathway, thereby suppressing its signaling activity [37]. Additionally, a study on ovarian cancer showed that fucoxanthin could inhibit the proliferation, migration, and invasion of ovarian cancer A2780 cells through the Akt/mTOR pathway [38]. MMP−2 and MMP−9, hydrolytic enzymes modulated by the PI3K/AKT signaling pathway, are often expressed at high levels in malignant tumors. Because MMP−2 and MMP−9 can degrade the extracellular matrix and promote tumor cell migration and invasion, they are considered essential for malignant invasion by and migration of tumor cells [39,40]. Overall, interactions between the PI3K signaling pathway and MMP−2/9 have substantial implications for tumor development and metastasis. These interactions could serve as targets for therapeutic strategies focused on inhibiting tumor invasion and metastasis. Therefore, suppressing the PI3K/AKT/mTOR signaling pathway and reducing the expression of MMP−2 and MMP−9 may be an effective therapeutic strategy to inhibit the metastasis of HPSCC cells. In this study, we demonstrated that fucoxanthin exerts its effects by suppressing the PI3K/AKT/mTOR pathway, leading to downregulation of MMP−2 and MMP−9 protein expression. Furthermore, we validated pathway protein expression, cell proliferation, migration, and invasion using the pathway activator 740 Y−P. Molecular docking analyses revealed that fucoxanthin can bind to all target proteins (PI3K, AKT, mTOR, MMP−2, and MMP−9). Fucoxanthin was able to form strong hydrogen bonding interactions with residues on these proteins; the short hydrogen bonding distance ensured stable binding within the complexes. These results suggest that fucoxanthin exerts its inhibitory effects on HPSCC proliferation and metastasis by regulating the expression of proteins in the PI3K/AKT/mTOR pathway, as well as the downstream proteins MMP−2 and MMP−9. Overall, fucoxanthin has potential for use in HPSCC treatment and could be developed into a clinically applicable drug in the future.

## 4. Materials and Methods

### 4.1. Cell Source and Culture

FaDu, Detroit 562, and 293T cells were purchased from the Shanghai Institute of Cell Biology, Chinese Academy of Sciences. Cells were grown in Dulbecco’s modified Eagle medium (DMEM) (Corning Inc., Corning, NY, USA), supplemented with 10% fetal bovine serum (Gibco, Los Angeles, CA, USA) and antibiotics (100 IU/mL penicillin and 100 g/mL streptomycin, TransGen, Beijing, China). Cells were maintained in a humidified environment at 37 °C with 5% CO_2_. Cells were passaged every other day to ensure optimal conditions for growth and proliferation.

### 4.2. Cell Viability and Observation of Cell Morphology

FaDu, Detroit 562, and 293T cells were seeded in 96-well plates (5 × 10^4^ cells/mL) and cultured overnight. The experimental group was treated with fucoxanthin (provided by Zhejiang Wanli University; purity > 95% as determined by high−performance liquid chromatography) for 48 h; the negative control group was treated with an equimolar mixture of dimethyl sulfoxide and DMEM for 48 h. Five replicate wells were prepared for each experimental condition. Cells were incubated at 37 °C for 4 h after the addition of MTT solution (Solarbio, Beijing, China). Culture medium was discarded and dimethyl sulfoxide was added; then, the absorbance at 570 nm was measured using a microplate reader (Allsheng, Hangzhou, China). FaDu and Detroit 562 cells were seeded in 6-well plates and cultured for 24 h. Subsequently, adherent cells were treated with fucoxanthin and allowed to proliferate for an additional 48 h. Cellular morphology was observed under a microscope (Nikon, Tokyo, Japan) at 100× magnification.

### 4.3. EdU Fluorescent Staining Assay

The proliferation abilities of FaDu and Detroit 562 cells treated with 740 Y−P (50 μg/mL) and various concentrations of fucoxanthin for 48 h were assessed using an EdU cell proliferation assay kit (Abbkine, Wuhan, China). EdU labeling was performed by adding pre-warmed EdU working solution to treated cells for 2 h. Culture medium was then removed; cells were fixed with 3.7% formaldehyde in phosphate-buffered saline and permeabilized with phosphate-buffered saline containing 0.5% Triton X-100 for 15 min each. Subsequently, 100 μL of click reaction solution was added to each well and cells were incubated at room temperature in the dark for 30 min. Finally, 100 μL of Hoechst 33342 solution was added to each well and cells were incubated at room temperature in the dark for 10 min. Fluorescence images were randomly captured at 200× magnification.

### 4.4. Cell Colony Formation Assay

Cells were seeded in 6-well plates (2 × 10^3^ cells/mL) and incubated for approximately 24 h to ensure attachment. Then, fucoxanthin treatment was initiated. The medium was replaced with fresh fucoxanthin-containing medium every 3 days. After 12 days, the culture medium was discarded; cells were fixed with 4% paraformaldehyde (Solarbio) and stained with 0.1% crystal violet (Solarbio). Next, they were washed with Dulbecco’s phosphate-buffered saline, air-dried, and photographed using a camera (Sony, Tokyo, Japan). The images were analyzed to determine the effects of fucoxanthin on colony formation.

### 4.5. Wound Healing Assay

FaDu and Detroit 562 cells were seeded in 6-well plates (2 × 10^5^ cells/mL) and incubated for approximately 24 h to reach 90% confluence. The tip of a pipette was used to create a longitudinal scratch on the cell monolayer. Cells were washed twice with phosphate-buffered saline, then treated with fucoxanthin and photographed (100× magnification) at 0, 24, and 48 h. Relative migration was quantified by measuring the healed scratch area compared with the initial 0-h time point.

### 4.6. Transwell Migration and Invasion Assays

Cell migration and invasion assays were conducted using Transwell chambers with 8-μm pore-size polycarbonate membranes in 6-well plates (Corning); the plates were precoated with Matrigel (Solarbio) for invasion assays. Cells were seeded in the upper chamber of the Transwell insert using serum-free medium containing 740 Y−P (50 μg/mL) and various concentrations of fucoxanthin; medium supplemented with 10% fetal bovine serum was placed in the lower chamber to serve as a chemoattractant. After 48 h of incubation at 37 °C with 5% CO_2_, cells were fixed with 4% paraformaldehyde. Cells on the underside of the Transwell membrane were stained with 0.1% crystal violet. Images were captured using an inverted microscope (Nikon), and cell numbers were recorded.

### 4.7. Cell Cycle Analysis

FaDu and Detroit 562 cells (5 × 10^4^ cells/mL) treated with fucoxanthin for 48 h were collected and fixed overnight at 4 °C using 70% ethanol. The fixation solution was then removed; cells were uniformly resuspended in PI/RNaseA solution and incubated for 15 min on a shaker at 4 °C in the dark. Finally, the cells were analyzed using a flow cytometer (FACSVerse, BD, Franklin Lakes, NJ, USA), and cell cycle analysis was performed using Modfit 5.0.9 software (Verity, Topsham, ME, USA).

### 4.8. Western Blotting Analysis

After fucoxanthin treatment, the total protein was recovered from cells using a protein extraction kit (Solarbio). Protein concentrations were measured and samples were normalized based on the measured values. The protein samples were then subjected to sodium dodecyl sulfate–polyacrylamide gel electrophoresis at 80 V for 2 h. Next, the proteins were transferred to nitrocellulose membranes at a voltage of 70 V. Subsequently, the membranes were incubated overnight with antibodies to the following proteins (all from ABclonal, Wuhan, China): CDK1 (1:1500), CDK2 (1:1500), CDK4 (1:1500), cyclin E1 (1:1500), PI3K (1:1500), AKT (1:1500), *p*−AKT (1:1500), mTOR (1:1500), *p*−mTOR (1:1500), MMP−2 (1:1500), MMP−9 (1:1500), and glyceraldehyde-3-phosphate dehydrogenase (GAPDH) (1:2000). Then, the membrane was incubated for 2 h with a secondary antibody (ABclonal, 1:4000), followed by immersion in an enhanced chemiluminescence reagent (EpiZyme Biomedical Technology, Shanghai, China) for 1 min. Gel imaging equipment was utilized to identify distinct protein bands, and grayscale values were analyzed using ImageJ 1.53t software (National Institutes of Health, Bethesda, MD, USA).

### 4.9. Molecular Docking

Molecular docking was performed to validate binding interactions between fucoxanthin and key target proteins. The PDB format files of PI3K (PDB ID: 4EZL), AKT (PDB ID: 3MVH), mTOR (PDB ID: 4JVE), MMP−2 (PDB ID: 8H78), and MMP−9 (PDB ID: 6ESM) proteins were downloaded from the Protein Data Bank (https://www.rcsb.org, accessed on 20 May 2024). Subsequently, the files were preprocessed in PyMOL software by removing crystallographic water molecules and ligands. Target proteins were prepared by adding hydrogen atoms in AutoDock 1.5.6; docking simulations were then performed with fucoxanthin. Docking was performed 20 times to ensure that the results were robust and reliable [41].

### 4.10. Statistical Analysis

Data were analyzed using GraphPad Prism 8, and the results are presented as means ± standard deviations. Unpaired *t*-tests were used to compare means between two groups; one-way analysis of variance was utilized for comparisons among ≥3 groups. Thresholds for statistical significance were regarded as * *p* < 0.05 and ** *p* < 0.01.

## 5. Conclusions

Fucoxanthin significantly inhibits the proliferation, migration, and invasion of HPSCC FaDu and Detroit 562 cells; it also inhibits cell cycle progression in those cells. Through reverse validation with pathway-specific activators, we found that these effects may be associated with downregulation of the PI3K/AKT/mTOR signaling pathway and its downstream proteins MMP−2 and MMP−9. Additionally, molecular docking results indicated that fucoxanthin can form hydrogen bonds and other interactions with PI3K, AKT, mTOR, MMP−2, and MMP−9, exhibiting high affinity with binding energies < −6 kcal/mol. Future studies should validate these findings in xenograft mouse models. Our work provides novel insights into the application of fucoxanthin as a functional food additive and establishes a foundation for the development of innovative therapeutic strategies targeting HPSCC.

## Data Availability

The data presented in this study are available from the corresponding authors upon request.

## Supplementary Materials

The following supporting information can be downloaded at: https://www.mdpi.com/article/10.3390/molecules29153603/s1. Figure S1: The effect of fucoxanthin on the viability of 293T cells after 24 and 48 h of treatment. Figure S2: Protein expression levels of AKT and mTOR in FaDu and Detroit 562 cells treated with various concentrations of fucoxanthin. Figure S3: Protein expression levels of AKT and mTOR in FaDu and Detroit 562 cells after combined treatment with 740 Y−P and fucoxanthin. Table S1: Hydrophobic interactions between fucoxanthin and PI3K. Table S2: Hydrogen bond interactions between fucoxanthin and PI3K. Table S3: Salt-bridge interactions between fucoxanthin and PI3K. Table S4: Hydrophobic interactions between fucoxanthin and AKT. Table S5: Hydrogen bond interactions between fucoxanthin and AKT. Table S6: Salt-bridge interactions between fucoxanthin and AKT. Table S7: Hydrophobic interactions between fucoxanthin and mTOR. Table S8: Hydrogen bond interactions between fucoxanthin and mTOR. Table S9: Salt-bridge interactions between fucoxanthin and mTOR. Table S10: Hydrophobic interactions between fucoxanthin and MMP−2. Table S11: Hydrogen bond interactions between fucoxanthin and MMP−2. Table S12: Hydrophobic interactions between fucoxanthin and MMP−9. Table S13: Hydrogen bond interactions between fucoxanthin and MMP−9.
