# Supplementary material for: Fucoxanthin Inhibits the Proliferation and Metastasis of Human Pharyngeal Squamous Cell Carcinoma by Regulating the PI3K/Akt/mTOR Signaling Pathway
Source: Molecules. 2024 Jul 30;29(15):3603. doi: 10.3390/molecules29153603 (PMC11314479; doi:10.3390/molecules29153603)
Supplement: Supplementary file 1 [file molecules-29-03603-s001.zip › molecules-3090172-supplementary.pdf]

Supplementary Materials:

1. Effect of fucoxanthin treatment on the cytotoxicity of 293T cells

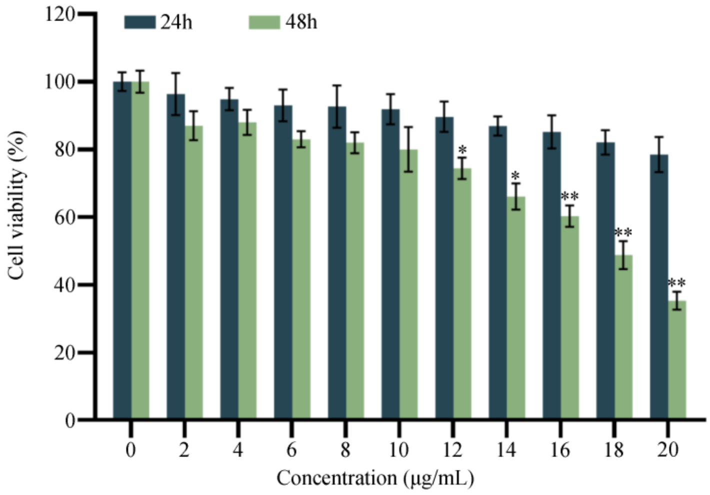

Figure S1. The effect of fucoxanthin on the viability of 293T cells after 24 and 48h of treatment.

2. Effect of fucoxanthin on AKT and mTOR protein expression levels in FaDu and Detroit 562 cells

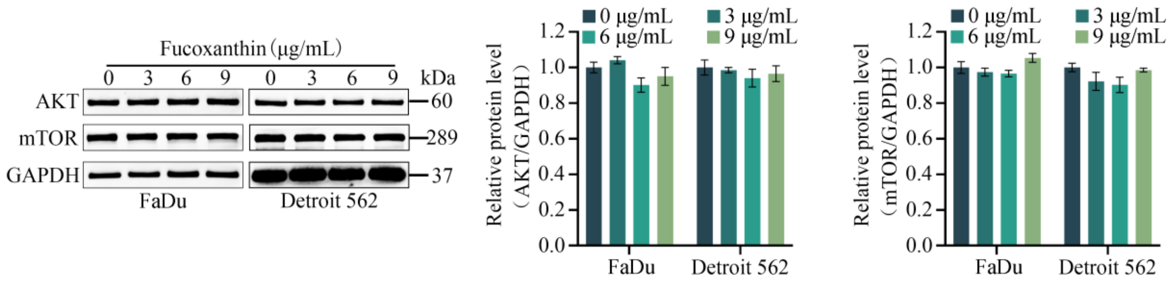

Figure S2. Protein expression levels of AKT and mTOR in FaDu and Detroit 562 cells treated with various concentrations of fucoxanthin.

3. Effect of 740 Y-P and fucoxanidin on protein expression levels of AKT and mTOR after FaDu and Detroit 562 cells

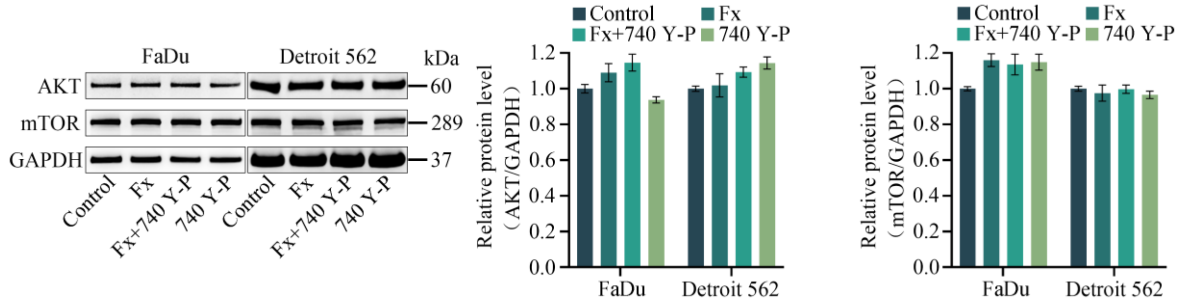

Figure S3. Protein expression levels of AKT and mTOR in FaDu and Detroit 562 cells after combined treat-ment with 740 Y-P and fucoxanthin.

5. Interaction between fucoxanthin and PI3K

Table S1. Hydrophobic interactions between fucoxanthin and PI3K.

Residue	AA	Distance	Ligand Atom	Protein Atom
940A	PHE	3.95	8251	6869
946A	ASP	3.53	8258	6913
982A	ARG	3.75	8254	7135
1031A	PHE	3.47	8269	7626
1035A	LEU	2.84	8270	7661
1040A	PRO	3.84	8275	7699
1042A	LEU	3.41	8275	7720
1050A	TYR	3.87	8264	7798
1050A	TYR	3.81	8261	7800

Table S2. Hydrogen bond interactions between fucoxanthin and PI3K.

Residue	AA	Distance H-A	Distance D-A	Donor Angle	Protein donor?	Side chine	Donor Atom	Acceptor Atom
983A	VAL	2.82	3.32	110.85	√	×	7147 [Nam]	8256 [O2]
1071A	GLN	1.99	2.92	150.30	√	√	8013 [Nam]	8292 [O3]
1071A	GLN	2.06	2.99	161.83	×	√	8292 [O3]	8016 [O2]

Table S3. Salt-bridge interactions between fucoxanthin and PI3K.

Residue	AA	Distance	Protein positive	Ligand Atom	Ligand Atom
947A	ARG	4.23	√	Carboxylate	8286, 8288

6. Interaction between fucoxanthin and AKT

Table S4. Hydrophobic interactions between fucoxanthin and AKT.

Residue	AA	Distance	Ligand Atom	Protein Atom
4B	ARG	3.97	3267	3192
156A	LEU	3.68	3285	122
164A	VAL	3.72	3290	200
164A	VAL	3.49	3288	202
164A	VAL	3.13	3287	203
164A	VAL	2.95	3300	201
179A	LYS	3.97	3300	351
234A	GLU	3.74	3278	913
236A	PHE	3.80	3276	935
236A	PHE	3.05	3274	937
236A	PHE	3.85	3269	938
237A	PHE	3.41	3278	944
438A	PHE	3.65	3285	2966
438A	PHE	3.20	3282	2964
442A	PHE	3.75	3304	3009

Table S5. Hydrogen bond interactions between fucoxanthin and AKT.

Residue	AA	Distance H-A	Distance D-A	Donor Angle	Protein donor?	Side chine	Donor Atom	Acceptor Atom
158A	LYS	2.04	2.74	123.88	√	×	131 [Nam]	3303 [O2]
240A	SER	2.77	3.51	135.09	√	√	987 [O3]	3271 [O2]
292A	ASP	2.22	3.15	164.21	×	√	3305 [O3]	1530 [O3]

Table S6. Salt-bridge interactions between fucoxanthin and AKT.

Residue	AA	Distance	Protein positive	Ligand Atom	Ligand Atom
4B	ARG	4.66	√	Carboxylate	3301, 3303

7. Interaction between fucoxanthin and mTOR

Table S7. Hydrophobic interactions between fucoxanthin and mTOR.

Residue	AA	Distance	Ligand Atom	Protein Atom
1417A	ILE	3.50	13624	325
1430A	ALA	3.34	13631	446
1453A	LEU	3.15	13646	677
1454A	HIS	3.41	13636	683
1583A	TYR	3.08	13612	1929

Table S8. Hydrogen bond interactions between fucoxanthin and mTOR.

Residue	AA	Distance H-A	Distance D-A	Donor Angle	Protein donor?	Side chine	Donor Atom	Acceptor Atom
1434A	GLU	1.94	2.82	150.28	×	√	13651 [O3]	478 [O2]

Table S9. Salt-bridge interactions between fucoxanthin and mTOR.

Residue	AA	Distance	Protein positive	Ligand Atom	Ligand Atom
2401A	HIS	4.88	√	Carboxylate	13647, 13649

8. Interaction between fucoxanthin and MMP-2

Table S10. Hydrophobic interactions between fucoxanthin and MMP-2.

Residue	AA	Distance	Ligand Atom	Protein Atom
5B	PHE	3.48	1591	45
6B	PRO	3.77	1600	57
6B	PRO	3.52	1590	56
9B	PRO	3.64	1591	87
83B	LEU	3.14	1564	813
84B	ALA	3.88	1564	822
85B	HIS	3.90	1569	828
87B	PHE	3.35	1578	850
121B	HIS	3.99	1560	1133

Table S11. Hydrogen bond interactions between fucoxanthin and MMP-2.

Residue	AA	Distance H-A	Distance D-A	Donor Angle	Protein donor?	Side chine	Donor Atom	Acceptor Atom
121B	HIS	1.71	2.61	145.68	√	√	1139 [Npl]	1567 [O2]
125B	HIS	2.34	3.17	137.57	√	√	1175 [Npl]	1567 [O2]
131B	HIS	2.29	3.24	153.98	√	√	1227 [Npl]	1567 [O2]
7B	ARG	2.20	3.20	121.30	√	√	1597 [O2]	1599 [O2]

9. Interaction between fucoxanthin and MMP-9

Table S12. Hydrophobic interactions between fucoxanthin and MMP-9.

Residue	AA	Distance	Ligand Atom	Protein Atom
179A	TYR	3.61	1561	688
187A	LEU	3.64	1575	779
187A	LEU	3.84	1592	768
189A	ALA	3.39	1581	789
190A	HIS	3.81	1572	795
192A	PHE	3.06	1565	817
192A	PHE	3.47	1561	819
208A	GLU	3.34	1587	951
210A	TRP	3.84	1587	979
218A	TYR	3.80	1585	1048
218A	TYR	3.32	1586	1050
218A	TYR	3.30	1587	1049
223A	VAL	3.17	1581	1099
223A	VAL	3.82	1582	1100
248A	TYR	3.56	1583	1319

Table S13. Hydrogen bond interactions between fucoxanthin and MMP-9.

Residue	AA	Distance H-A	Distance D-A	Donor Angle	Protein donor?	Side chine	Donor Atom	Acceptor Atom
179A	TYR	2.08	2.70	120.17	×	√	1604 [O3]	690 [O3]
248A	TYR	2.28	3.00	126.53	√	×	1314 [Nam]	1602 [O3]
248A	TYR	3.35	3.79	110.03	×	×	1602 [O3]	1318 [O2]
